# Bioinformatic analysis of the role of immune checkpoint genes and immune infiltration in the pathogenesis and development of premature ovarian insufficiency

**DOI:** 10.1007/s10815-024-03120-x

**Published:** 2024-05-02

**Authors:** Xiyan Zhang, Ling Wang, Tongkun Yang, Li Kong, Luxiao Wei, Jing Du

**Affiliations:** 1 The 940, Hospital of Joint Logistic Support Force of Chinese People’s Liberation Army, Gansu, 730050 China; 2 Department of Obstetrics and Gynecology the First Medical Center of Chinese, PLA General Hospital, Beijing, 100039 China; 3 Gansu University of Chinese Medicine, Gansu, 730030 China

**Keywords:** Gene Expression Omnibus database, Premature Ovarian Insufficiency, Immune checkpoint genes, Bioinformatics

## Abstract

**Purpose:**

With advances in immunology, increasing evidence suggests that immunity is involved in premature ovarian insufficiency (POI) pathogenesis. This study investigated the roles of immune checkpoint genes and immune cell infiltration in POI pathogenesis and development.

**Methods:**

The GSE39501 dataset and immune checkpoint genes were obtained from the Gene Expression Omnibus database and related literature. The two datasets were intersected to obtain immune checkpoint-related differentially expressed genes (ICRDEGs), which were analyzed using Gene Ontology and Kyoto Encyclopedia of Gene and Genomes enrichment analysis, weighted correlation network analysis, protein–protein interaction and related microRNAs, transcription factors, and RNA binding proteins. The immune cell infiltration of ICRDEGs was explored, and receiver operating characteristic curves were used to validate the diagnostic value of ICRDEGs in POI.

**Results:**

We performed ICRDEG functional enrichment analysis and found that these genes were closely related to immune processes, such as T cell activation. Specifically, they are enriched in various biological processes and pathways, such as cell adhesion molecule and T cell receptor signaling pathways. Weighted correlation network analysis identified seven hub genes: *Cd200*, *Cd274*, *Cd28*, neurociliary protein-1, *Cd276*, *Cd40lg*, and *Cd47*. Furthermore, we identified 112 microRNAs, 17 RNA-binding proteins, and 101 transcription factors. Finally, immune infiltration analysis showed a clear positive correlation between hub genes and multiple immune cell types.

**Conclusion:**

Bioinformatic analysis identified seven potential ICRDEGs associated with POI, among which the immune checkpoint molecules *CD200* and neurociliary protein-1 may be involved in the pathogenesis of POI.

**Supplementary Information:**

The online version contains supplementary material available at 10.1007/s10815-024-03120-x.

## Introduction

POI is defined as amenorrhea with high gonadotropin and low estrogen levels in women younger than 40 years [1]. The cessation of menses at a younger age leads to infertility and decline in psychological well-being and long-term quality of life of these patients. The pathogenesis of POI is diverse, including iatrogenic factors (post-surgery, radiotherapy, or chemotherapy), autoimmune ovarian function impairment, environmental factors (viruses, chemical agents, and radiation), metabolic diseases (diabetes type 1, galactosemia, 17-OH deficiency, and 21-OH deficiency), and unexplained factors [2]. Given the absence of an effective method to restore ovarian function in POI [3], it is necessary to identify markers of POI for earlier identification and intervention.

The immune system is composed of several relevant molecular and chemical systems, their primary function is to protect the body from pathogens. The immune system plays multiple roles in the female reproductive system, mainly in gonadal activity and regulation of the female reproductive tract, and ovarian cycle [4]. Disease may occur if normal physiological interactions between the immune and reproductive systems are dysregulated.

Immune checkpoint molecules exist on the surface of immune cells, and the negative regulation of effector immune cells prevents the body from being attacked by its own antigens [5]. The PD-1 immune checkpoint pathway is involved in follicle formation and ovulation [6, 7]. Little is known about the normal interactions between female germ and immune cells, necessitating this relationship to be characterized.

Based on bioinformatics analysis, this study explored the possible pathophysiological process of ovarian disease from the perspective of immune checkpoint molecules related to POI based on the Weighted Gene Association Network Analysis (WGCNA) and immune infiltration. Further studies on the relation between POI and immune checkpoint genes, as well as immune cell infiltration, could provide new insights into the immune mechanisms associated with POI.

## Materials and Methods

### Data resource

We used “premature ovarian insufficiency” and “premature ovarian failure” as keyword on the Gene Expression Omnibus(GEO) database, microarray datasets were obtained with the accession no [8, 9]. Dataset GSE39501, obtained from *Mus musculus*, comprised samples from patients with POI and normal samples, totaling six samples. The data platform file used was the GPL6887 Illumina MouseWG-6 v2.0 expression beadchip. We included the expression profile data of three samples from patients with POI (POI group) and three normal samples (Normal group). The details of the dataset are listed in Table 1.

**Table 1:** Premature ovarian insufficiency (POI) dataset information

Species	Mus musculus
Samples in Normal group	3
Samples in POI group	3
GPL	GPL6887
Reference	[8]

POI: premature ovarian insufficiency

The published literatures included in PubMed database were searched with the key words “immune checkpoint genes”, and finally we identified 73 immune checkpoint genes for analysis [10] (see Table S1).

### Differentially expressed genes (DEGs) associated with POI

We performed differential analysis of dataset GSE39501 using the softwear package in R used for analysis of differential gene expression [11] to obtain DEGs among the Normal and POI groups, and genes with |logFC|> 0 and P-value < 0.05 were selected as DEGs for further study. Genes with logFC > 0 and P-value < 0.05 were DEGs (upregulated genes), and those with logFC < 0 and P-value < 0.05 were DEGs (downregulated genes).

To obtain the immune checkpoint-related DEGs (ICRDEGs) associated with POI, we crossed the DEGs in the GSE39501 dataset with immune checkpoint-related genes and drew a Venn diagram, the results of the differential analysis were presented in a volcano map and heat map using the R package ggplot2.

### Functional and pathway enrichment analyses of DEGs

We performed Gene Ontology (GO) [12] and Kyoto Encyclopedia of Genes and Genomes (KEGG) [13] annotation analysis of ICRDEGs using the R package clusterProfiler [14]. Entry screening criteria of P-value < 0.05 and FDR value (q.value) < 0.05 were considered statistically significant, with P-value correction applied using the Benjamini–Hochberg method.

### Gene Set Enrichment Analysis (GSEA)

We obtained data from the Molecular Signatures Database “m2.all.v2022.1” gene set, specifically using the “Mm.symbols.gmt” gene set. GSEA [15] was performed with the expressed genes in the GSE39501 dataset, with the screening criteria P-value < 0.05 and FDR value (q.value) < 0.25 indicating significant enrichment.

### Gene Set Variation Enrichment Analysis (GSVA)

We obtained the “m2.all.v2022.1” gene set from Molecular Signatures Database. GSVA [16] was used to analyze the GSE39501 dataset for calculating the difference in functional enrichment between the Normal and POI groups.

### WGCNA

We performed WGCNA [17] on the GSE39501 dataset using the R package WGCNA. Selected genes with |logFC|> 0 and P-value < 0.05 were inputted in the WGCNA. The minimum number of module genes was set to 100, and the softpower was optimized with a soft threshold of 14. The combined shear height of the modules was set to 0.2, and the minimum distance was set to 0.2 to assess the correlation of the Normal and POI groups with different modules. Genes in each module were recorded, and module eigengenes were considered. After selecting the modules of interest based on the correlation values, we identified all genes within the module as DEGs that are highly associated with POI.

### Immune infiltration analysis

In this study, the immune cell infiltration status in the GSE39501 dataset was evaluated using the CIBERSORT (https://cibersort.stanford.edu/) [18] algorithm. Spearman correlation was employed to assess the correlation between various immune cells, and thus, determine the correlation between genes with high correlation to candidate and immune infiltrating cells. The correlation between immune cells and ICRDEGs combined with the gene expression matrix of the POI dataset was then assessed, and the correlation heat map was drawn using the R package pheatmap.

The enrichment scores calculated by the single-sample GSEA (ssGSEA) [19] algorithm in the R package GSVA were used to represent the infiltration level of each immune cell type in each sample, and the correlation between the immune infiltrating cells was determined by Spearman correlation analysis.

For Spearman correlation analysis based on MCPcounter abundance estimates as well as expression of antigen genes, a P-value < 0.05 was considered statistically significant.

### Protein–protein interaction (PPI) networks and mRNA-microRNA (miRNA) mRNA-RNA binding protein (RBP) mRNA-transcription factor (TF) interaction networks

In this study, we used the STRING database [20] to screen the ICRDEGs selected from the differential analysis to construct PPI networks associated with DEGs and to visualize the PPI network model using Cytoscape. The closely linked local regions of the PPI network, which may represent molecular complexes, have specific biological functions.

The ENCORI [21] (https://starbase.sysu.edu.cn/) and miRDB databases [22] were used for miRNA target gene prediction and functional annotation. We used the ENCORI and miRDB databases to predict the miRNAs interacting with hub genes (mRNA) and then utilized the intersection part of the results of the two databases to draw the mRNA-miRNA interaction network.

We searched for TFs bound to hub genes (mRNA) using the CHIPBase database [23] (version 3.0; https://rna.sysu.edu.cn/chipbase/).

### Correlation analysis and receiver operating characteristic curve analysis of DEGs

The pROC package was used to plot the receiver operating characteristic curves of hub genes in the Normal and POI groups in the two datasets, and the area under the curve was calculated to evaluate the diagnostic value of hub gene expression for POI.

### Statistical analysis

All data processing and analyses in this study were performed using R software (Version 4.1.2). For the comparison of two continuous variables, the statistical significance of normally distributed variables was estimated using the independent Student’s t-test, and differences between non-normally distributed variables were analyzed using the Mann–Whitney U test (i.e., Wilcoxon rank sum test). Chi-square or Fisher's exact tests were used to compare and analyze the statistical significance between two groups of categorical variables. If not explicitly specified, the results were calculated as correlation coefficients between different molecules by Spearman correlation analysis, and all P-values were two-sided, with P < 0.05 considered statistically significant.

## Results

### Technical roadmap

#### Data standardization

**Fig. 1:**
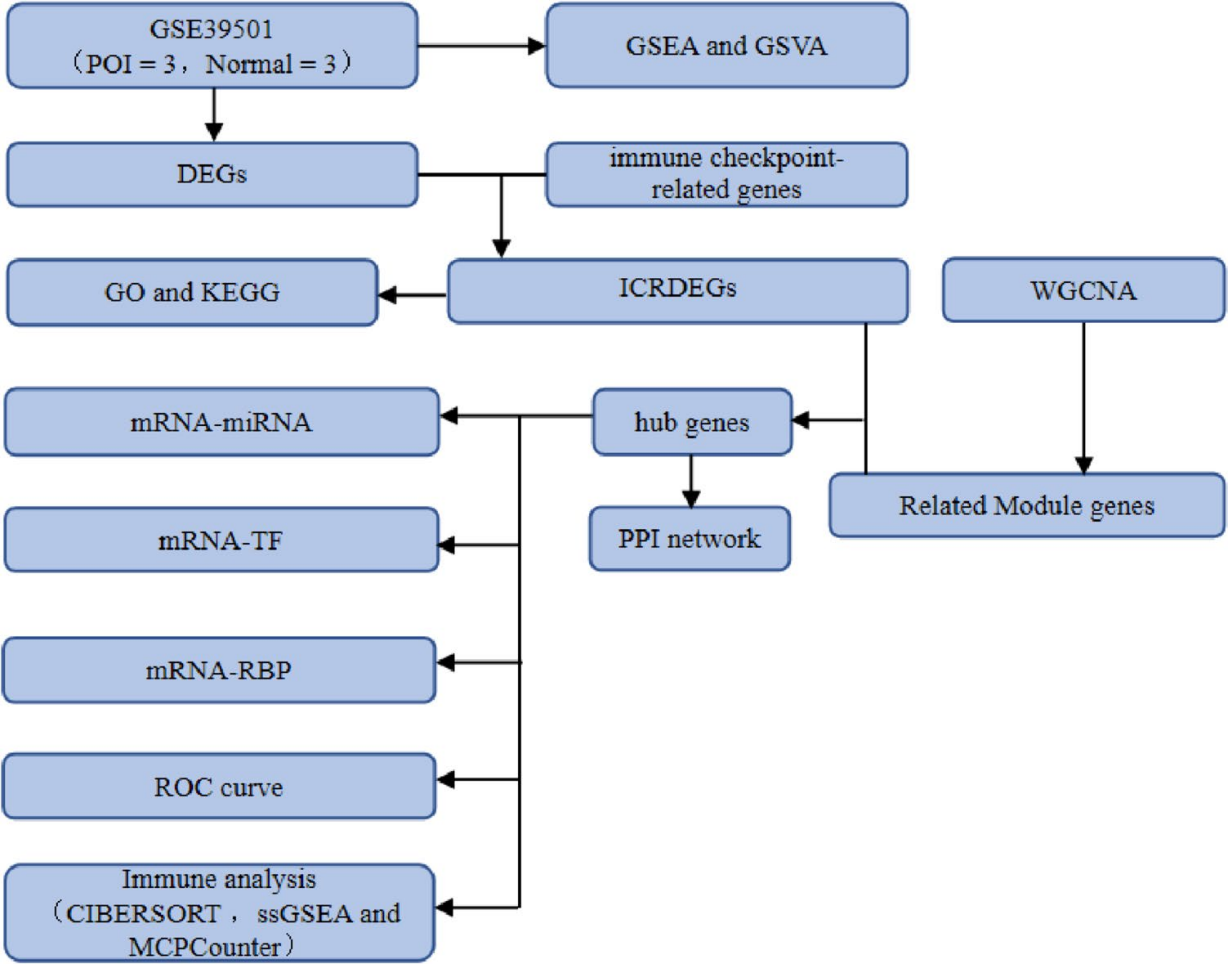
Technical Roadmap. POI: Premature ovarian insufficiency. GSEA: Gene Set Enrichment Analysis. GSVA: Gene Set Variation Analysis. ssGSEA: single-sample Gene Set Enrichment Analysis. GO: Gene Ontology. KEGG: Kyoto Encyclopedia of Genes and Genomes. PPI network: Protein–Protein Interaction network. ROC: Receiver Operating Characteristic PCA: Principal Component Analysis. ICRDEGs: immune checkpoint-related differentially expressed genes. DEGs: Differentially Expressed Senes

**Fig. 2:**
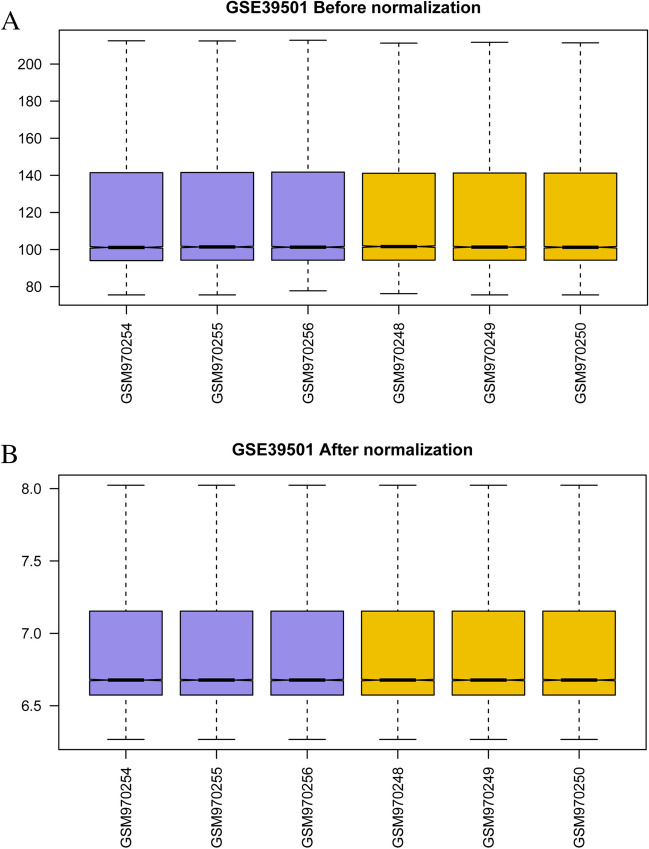
Boxplot before and after correction of the GSE39501 dataset for POI. **A**. Box plot of gene expression distribution between samples in the GSE39501 dataset before correction. **B**. Box plot of gene expression distribution among samples for the corrected GSE39501 dataset. Yellow represents the normal group and purple represents the POI sample group. POI: Premature ovarian insufficiency

This study mainly explored the biological characteristics of POI using bioinformatics methods, and a flow chart of the overall analysis is shown in Fig. 1. In the GSE39501 dataset, the samples were divided into the POI and Normal groups. Subsequently, the GSE39501 dataset (Fig. 2A–B) was standardized, data cleaning operations, such as annotated probes, were performed, and the data distribution before and after standardization was boxed. After normalization, the expression trends converged across different samples in the dataset.

### Analysis of DEGs associated with POI

**Fig. 3:**
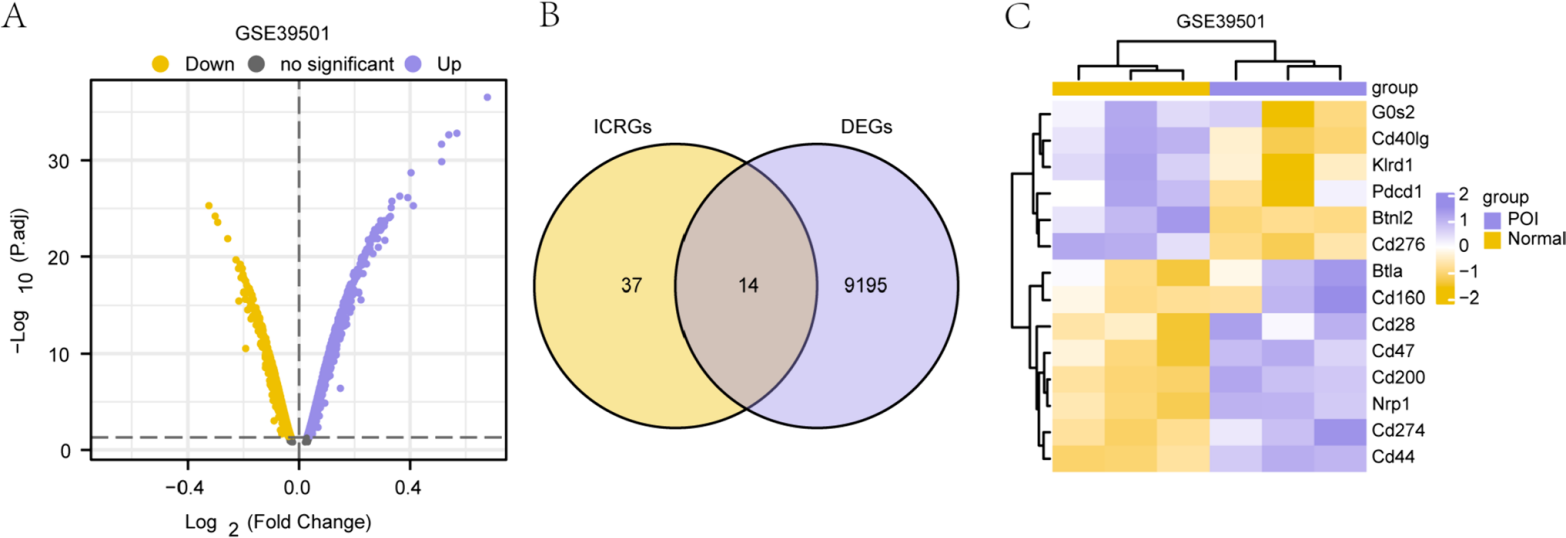
Differential gene analysis of the premature ovarian insufficiency dataset. **A**. Volcano plots of differential genes in the GSE39501 dataset. **B**. Venn diagram of the DEGs and ICRGs in the GSE39501 dataset. **C**. Heat map of differentially expressed genes in the GSE39501 dataset. DEGs: differentially expressed genes. ICRGs: immune checkpoint-related genes

There were 9209 DEGs in total, including 4309 genes being up-regulated, and 4900 genes being down-regulated. We visualized the results with a volcano map based on the differential analysis of this dataset (Fig. 3A).

To obtain ICRDEGs, we took the intersection of DEGs and immune checkpoint-related genes with |logFC|> 0 and P-values < 0.05 obtained in the GSE39501 dataset. This process yielded 14 ICRDEGs. The results were visualized using a Venn diagram (Fig. 3B).

We also analyzed the differences in expression between POI and Normal groups in the GSE39501 dataset (Fig. 3C) and used the R package pheatmap to generate a heat map illustrating the expression patterns of the 14 identified ICRDEGs (*Btla*, *Btnl2*, *Cd160*, *Cd200*, *Cd274*, *Cd276*, *Cd28*, *Cd40lg*, *Cd44*, *Cd47*, *G0s2*, *Klrd1*, neurociliary protein-1 [*Nrp1*], and *Pdcd1*). The results showed that 14 DEGs clustered significantly in the two groups within the GSE39501 dataset.

### Functional and pathway enrichment analyses of ICRDEGs

**Fig. 4:**
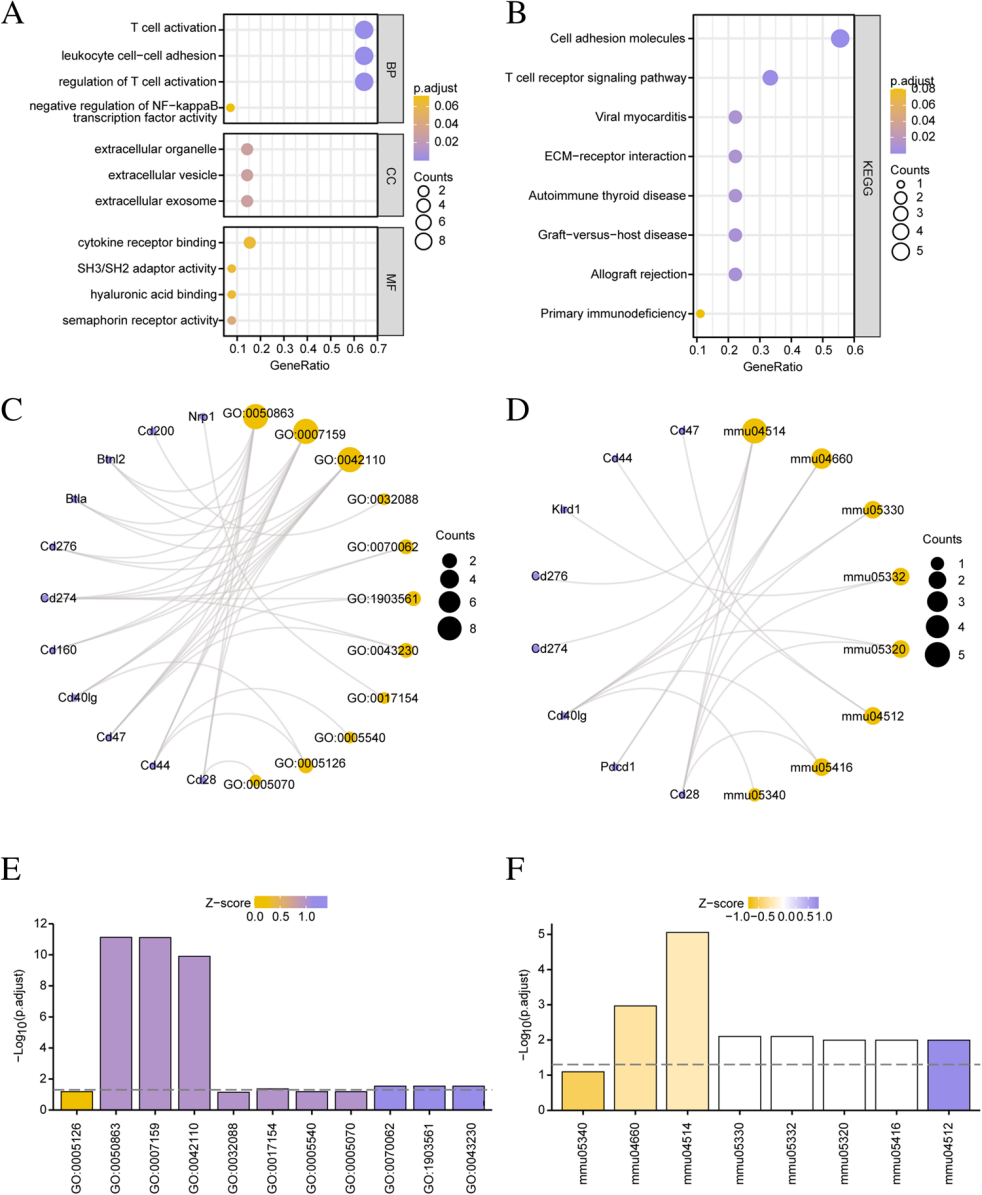
Functional enrichment analysis (GO) and pathway enrichment (KEGG) analysis of ICRDEGs. **A–B**. Bubble diagram display of GO function enrichment analysis (**A**) and KEGG pathway enrichment analysis (**B**) of ICRDEGs. **C–D**. Ring network diagram display of GO function enrichment analysis (**C**) and KEGG pathway enrichment analysis (**D**) of ICRDEGs. E–F. GO function enrichment analysis (**E**) and KEGG pathway enrichment analysis (**F**) of ICRDEGs. ICRDEGs: immune checkpoint-related differentially expressed genes. GO: Gene Ontology. BP: Biological Orocess. CC: Cellular Component. MF: Molecular Function. KEGG: Kyoto Encyclopedia of Genes and Genomes. The screening criteria for the GO and KEGG enrichment entries were P-value < 0.05 and FDR value (q.value) < 0.05

To analyze the biological processes associated with the 14 ICRDEGs along with their molecular function, cellular component, biological pathways, and relation with POI, we first performed GO (Table 2) and KEGG (Table 3) enrichment analysis for ICRDEGs. The screening criterion for enrichment entries was defined as P-value < 0.05, with an associated FDR value (q.value) < 0.05, considered statistically significant. The results of GO functional enrichment analysis and KEGG enrichment analysis are displayed as a bubble map (Fig. 4A–B) and a ring network map (Fig. 4C–D). Subsequently, we showed the results of GO function enrichment analysis of ICRDEGs combined with logFC (Fig. 4E–F).

**Table 2:** GO enrichment analysis results of ICRDEGs

ONTOLOGY	ID	Description	GeneRatio	BgRatio	pvalue	p.adjust	qvalue
BP	GO:1903037	regulation of leukocyte cell–cell adhesion	9/14	287/23210	1.13e-14	7.44e-12	2.72e-12
BP	GO:0050863	regulation of T cell activation	9/14	304/23210	1.91e-14	7.44e-12	2.72e-12
BP	GO:0007159	leukocyte cell–cell adhesion	9/14	319/23210	2.95e-14	7.67e-12	2.80e-12
BP	GO:1903039	positive regulation of leukocyte cell–cell adhesion	8/14	203/23210	8.56e-14	1.67e-11	6.10e-12
BP	GO:0022407	regulation of cell–cell adhesion	9/14	395/23210	2.03e-13	3.17e-11	1.16e-11
CC	GO:0070062	extracellular exosome	2/14	90/23436	0.001	0.029	0.023
CC	GO:1903561	extracellular vesicle	2/14	98/23436	0.002	0.029	0.023
CC	GO:0043230	extracellular organelle	2/14	113/23436	0.002	0.029	0.023
CC	GO:0005883	neurofilament	1/14	10/23436	0.006	0.064	0.050
CC	GO:0005769	early endosome	2/14	263/23436	0.010	0.090	0.070
MF	GO:0023023	MHC protein complex binding	2/13	12/22710	1.99e-05	7.56e-04	4.40e-04
MF	GO:0098632	cell–cell adhesion mediator activity	2/13	30/22710	1.30e-04	0.002	0.001
MF	GO:0098631	cell adhesion mediator activity	2/13	39/22710	2.21e-04	0.003	0.002
MF	GO:0005539	glycosaminoglycan binding	2/13	208/22710	0.006	0.043	0.025
MF	GO:1990405	protein antigen binding	1/13	11/22710	0.006	0.043	0.025

ICRDEGs: immune checkpoint-related differentially expressed genes. GO: Gene Ontology. BP: Biological Process. CC: Cellular Component. MF: Molecular Function

**Table 3:** KEGG enrichment analysis results of ICRDEGs

ONTOLOGY	ID	Description	GeneRatio	BgRatio	*P*-value	p.adjust	q.value
KEGG	mmu04514	Cell adhesion molecules	5/9	175/8910	3.26e-07	8.81e-06	5.15e-06
KEGG	mmu05235	PD-L1 expression and PD-1 checkpoint pathway in cancer	3/9	88/8910	7.49e-05	0.001	5.91e-04
KEGG	mmu04660	T cell receptor signaling pathway	3/9	103/8910	1.20e-04	0.001	6.31e-04
KEGG	mmu04672	Intestinal immune network for IgA production	2/9	43/8910	8.02e-04	0.005	0.003
KEGG	mmu05330	Allograft rejection	2/9	64/8910	0.002	0.008	0.005

ICRDEGs: immune checkpoint related differentially expressed genes. KEGG: Kyoto Encyclopedia of Genes and Genomes

The results of these analyses showed that the genes were mainly enriched in the regulation immunity and inflammation biological process(GO:0032088). For the cellular component, the gene were mainly enriched the carriers of biomarkers in extracellular spaces(GO:0070062). Finally, regarding molecular function, the genes were mainly enriched in the receptor function(GO:0017154). Furthermore, KEGG enrichment analysis revealed that the ICRDEGs were mainly associated with pathways, such as cell adhesion molecules (mmu04514), T cell receptor signaling pathway (mmu04660), allograft rejection (mmu05330), graft-versus-host disease (mmu05332), autoimmune thyroid disease (mmu05320), extracellular matrix-receptor interaction (mmu04512), viral myocarditis (mmu05416), and primary immunodeficiency (mmu05340).

**Fig. 5:**
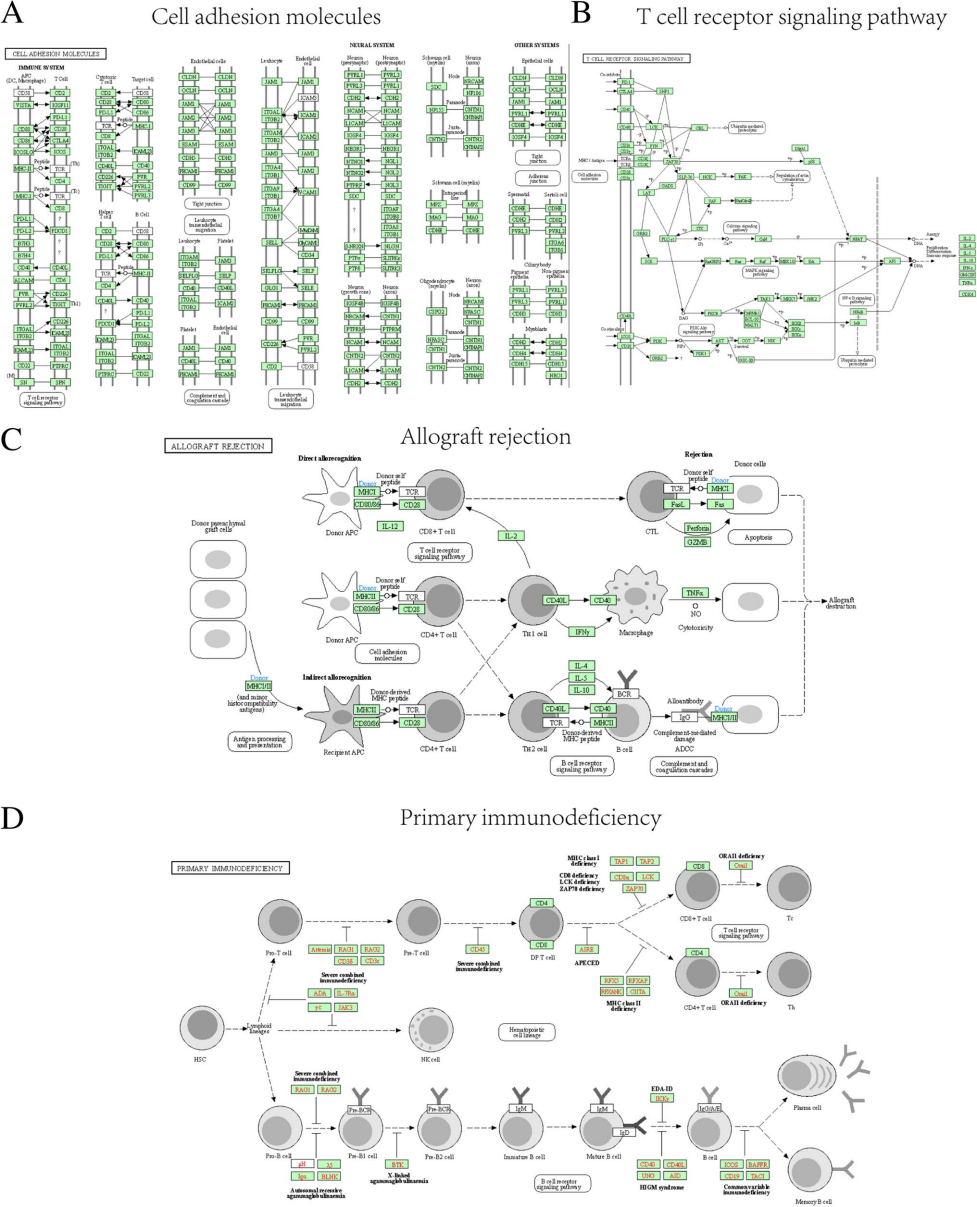
Pathway enrichment (KEGG) analysis of ICRDEGs. **A–D** Pathway enrichment (KEGG) enrichment analyses results for ICRDEGs. KEGG pathway cell adhesion molecules (**A**), T cell receptor signaling pathway (**B**), allograft rejection (**C**), and primary immunodeficiency (**D**). ICRDEGs: immune checkpoint-related differentially expressed genes. KEGG: Kyoto Encyclopedia of Genes and Genomes

We used the R package Pathview (Fig. 5) to illustrate the results for cell adhesion molecules (Fig. 5A), T cell receptor signaling pathway (Fig. 5B), allograft rejection (Fig. 5C), primary immunodeficiency (Fig. 5D), and four KEGG pathways in the KEGG enrichment analysis.

### GSEA and GSVA enrichment analysis of the POI dataset

**Fig. 6:**
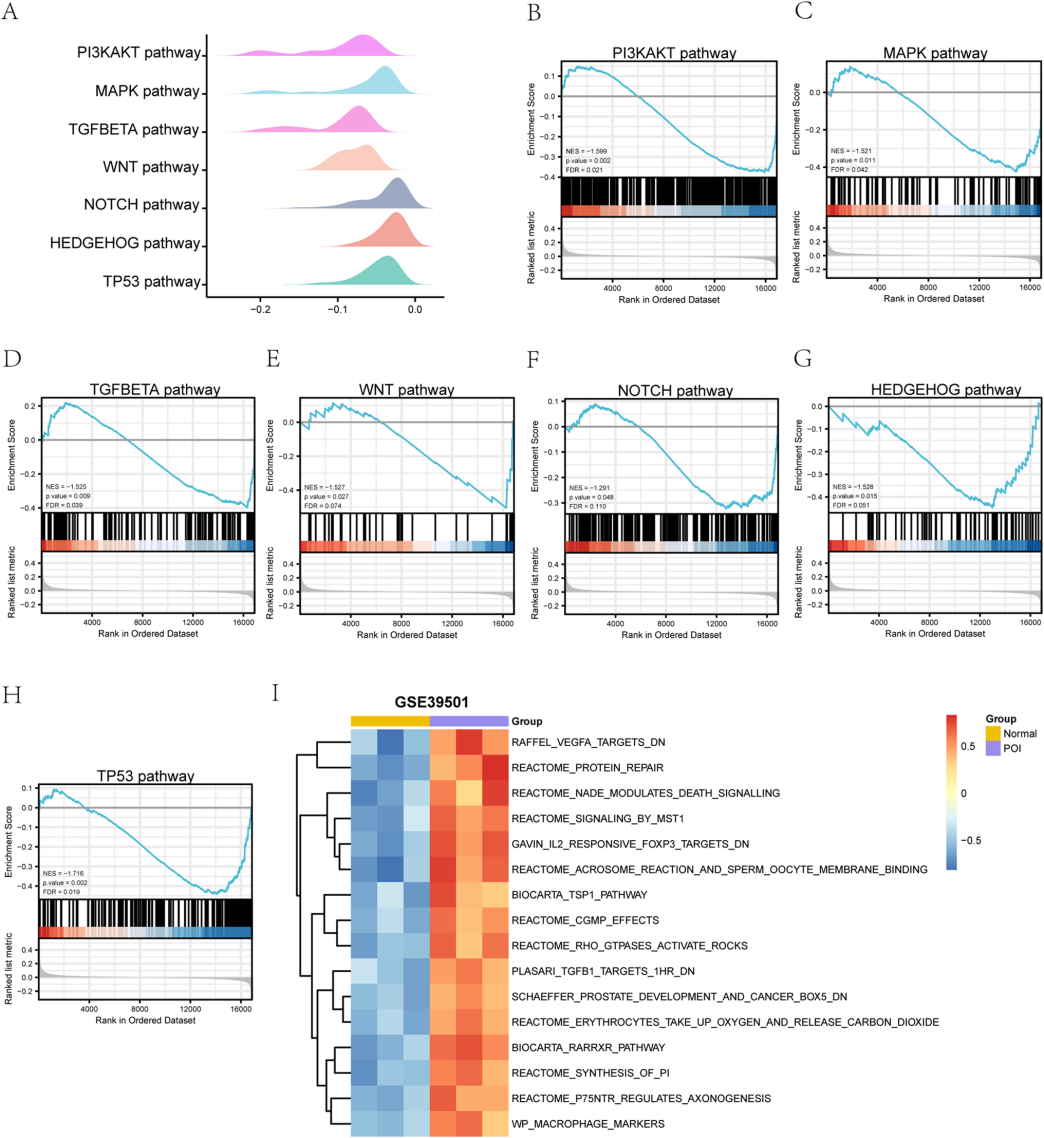
GSEA and GSVA enrichment analysis for the GSE39501 Dataset. **A**. GSEA enrichment analysis of the GSE39501 dataset. B–H. Differential genes in the GSE39501 dataset were significantly enriched in the PI3K-AKT pathway (**B**), MAPK pathway (**C**), TGFBETA pathway (**D**), WNT pathway (**E**), NOTCH pathway (**F**), HEDGEHOG pathway (**G**), and TP53 pathway (**H**). I. GSVA analysis of the GSE39501 dataset. Yellow represents the Normal group and purple represents the POI group. POI: premature ovarian insufficiency. GSEA: Gene Set Enrichment Analysis. GSVA: Gene Set Variation Analysis. The screening criteria for determining significant enrichment in GSEA and GSVA enrichment analyses were P-value < 0.05 and FDR value (q.value) < 0.25

To determine the effect of gene expression levels on POI, we analyzed the links between gene expression in the Normal and POI groups and biological processes, affected cellular components, and molecular functions using GSEA enrichment analysis. All genes in the GSE39501 dataset were significantly enriched in the phosphatidylinositol 3-kinase (PI3K)-AKT pathway (Fig. 6B), MAPK pathway (Fig. 6C), TGFbeta pathway (Fig. 6D), WNT pathway (Fig. 6E), NOTCH pathway (Fig. 6F), HEDGEHOG pathway (Fig. 6G), TP53 pathway (Fig. 6H), and other pathways (Table 4).

**Table 4:** GSEA analysis of GSE39501

Description	setSize	enrichmentScore	NES	*P*-value	p.adjust	q.values
WP_PI3KAKT_SIGNALING_PATHWAY	303	-0.376173126	-1.598679568	0.002932551	0.028937013	0.02073761
BIOCARTA_MAPK_PATHWAY	79	-0.425258851	-1.521016048	0.011363636	0.059096028	0.042350963
WP_TGFBETA_SIGNALING_PATHWAY	124	-0.397233262	-1.525287822	0.00973236	0.055078479	0.039471801
ST_WNT_BETA_CATENIN_PATHWAY	34	-0.505713349	-1.526616872	0.027484144	0.102594864	0.073524253
REACTOME_SIGNALING_BY_NOTCH	171	-0.32276312	-1.290532187	0.048	0.152871724	0.109554991
REACTOME_HEDGEHOG_LIGAND_BIOGENESIS	62	-0.446509357	-1.527778106	0.015384615	0.070622419	0.050611312
REACTOME_REGULATION_OF_TP53_ACTIVITY	137	-0.441052681	-1.716255162	0.002475248	0.026832613	0.019229499
KEGG_VIRAL_MYOCARDITIS	54	0.627075344	1.986349836	0.001801802	0.026832613	0.019229499
KEGG_LEISHMANIA_INFECTION	61	0.624971251	2.014187572	0.001808318	0.026832613	0.019229499
KEGG_DRUG_METABOLISM_CYTOCHROME_P450	59	0.551351776	1.767894742	0.001811594	0.026832613	0.019229499
REACTOME_INTERFERON_GAMMA_SIGNALING	71	0.634411829	2.085915206	0.001814882	0.026832613	0.019229499
REACTOME_COMPLEMENT_CASCADE	43	0.714853598	2.182780888	0.001824818	0.026832613	0.019229499
KEGG_ANTIGEN_PROCESSING_AND_PRESENTATION	51	0.628595144	1.972941257	0.001834862	0.026832613	0.019229499
KEGG_SYSTEMIC_LUPUS_ERYTHEMATOSUS	52	0.715392314	2.247654932	0.001834862	0.026832613	0.019229499
REACTOME_ANTIMICROBIAL_PEPTIDES	51	0.683319413	2.14470168	0.001834862	0.026832613	0.019229499

GSEA: Gene Set Enrichment Analysis

To explore the differences in the GSE39501 dataset between the POI samples and the corresponding normal samples, we performed GSVA enrichment analysis (Fig. 6I) and assessed the differences in functional enrichment. The results showed that the IL-2 pathway isogene set showed differences between the POI and Normal groups (Table 5).

**Table 5:** GSVA analysis of the GSE39501 dataset

	logFC	AveExpr	t	*P*-value	adj.P.Val
GAVIN_IL2_RESPONSIVE_FOXP3_TARGETS_DN	1.236872591	0.025409892	9.520929316	2.91E-05	0.001366311
BIOCARTA_RARRXR_PATHWAY	1.205013925	0.042861334	9.392266038	3.18E-05	0.001366311
REACTOME_SYNTHESIS_OF_PI	1.12142358	-0.011477326	8.90115136	4.52E-05	0.001366311
REACTOME_PROTEIN_REPAIR	1.243469862	-0.022293938	8.777474541	4.95E-05	0.057411212
REACTOME_ACROSOME_REACTION_AND_SPERM_OOCYTE_MEMBRANE_BINDING	1.288114651	-0.007619104	8.701379223	5.24E-05	0.124694938
REACTOME_RHO_GTPASES_ACTIVATE_ROCKS	1.111268476	-0.003744006	8.542065419	5.91E-05	0.124694938
REACTOME_ERYTHROCYTES_TAKE_UP_OXYGEN_AND_RELEASE_CARBON_DIOXIDE	1.043380907	-0.015671294	8.520553726	6.00E-05	0.150420308
REACTOME_P75NTR_REGULATES_AXONOGENESIS	1.072790364	-0.010737151	8.511929511	6.04E-05	0.21282653
SCHAEFFER_PROSTATE_DEVELOPMENT_AND_CANCER_BOX5_DN	1.007028576	-0.043604521	8.187051178	7.76E-05	0.272935559

GSVA: Gene Set Variation Analysis

### WGCNA analysis to identify the co-expression module in the GSE39501 dataset

**Fig. 7:**
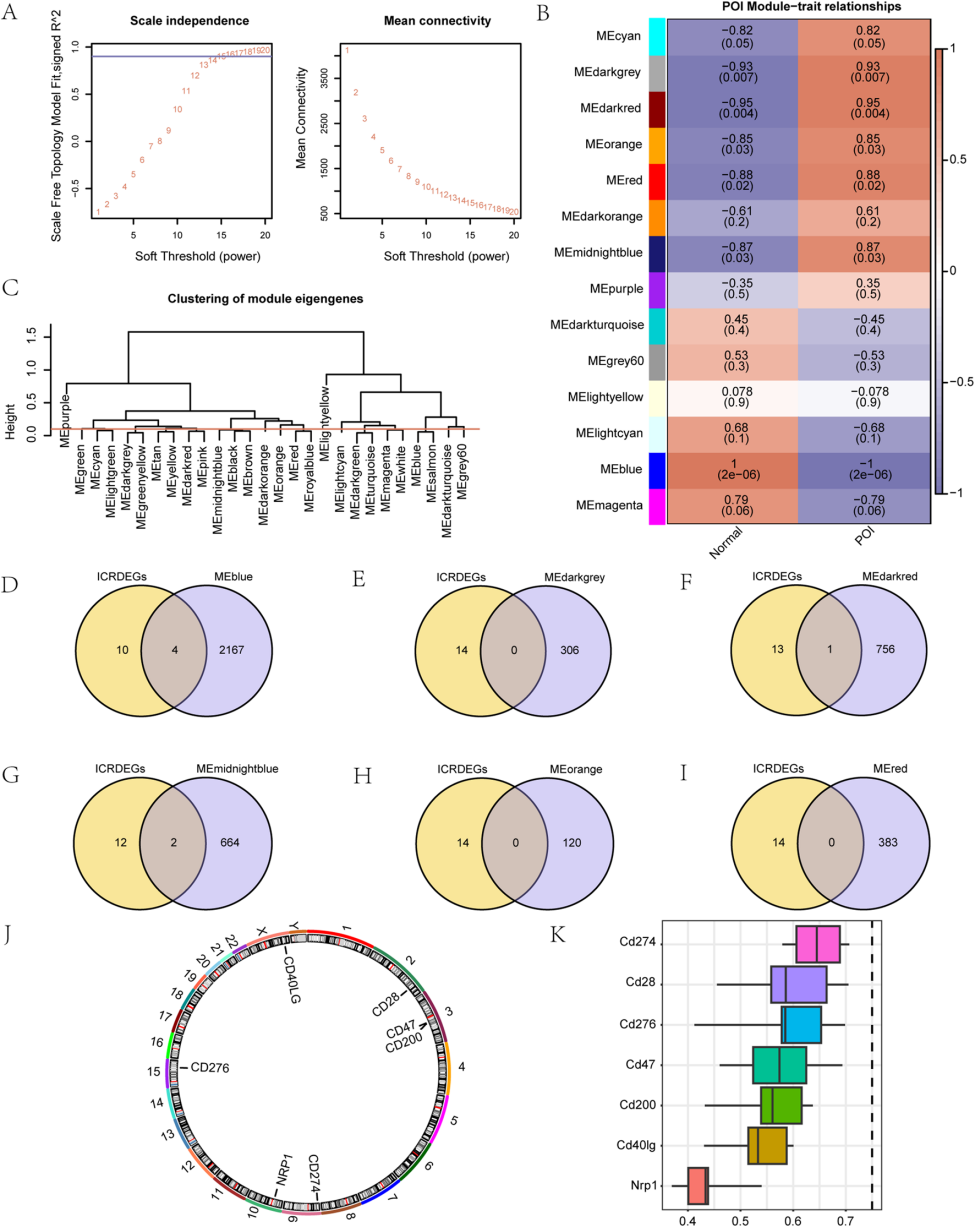
WGCNA analysis identifies the co-expression module in the GSE39501 dataset. **A**. Sample module screening threshold in the GSE39501 dataset. **B**. Correlation analysis between clustering modules of DEGs and different groups in the GSE39501 dataset. **C**. Results of DEGs in the GSE39501 dataset. D-I. DEGs in the GSE39501 dataset shown in Venn diagrams. MEblue (**D**), MEdarkgrey (**E**), MEdarkred (**F**), MEmidnightblue (**G**), MEorange (**H**), and MEred (**I**). J. Chromosomal localization map of hub genes. K. Visual presentation of hub genes Friends analysis. WGCNA: Weighted Gene Association Network Analysis. POI: Premature Ovarian Insufficiency. DEGs: Differentially Expressed Genes. ICRDEGs: immune checkpoint-related differentially expressed genes

We performed a WGCNA of all |logFC|-expressed genes (DEGs) in the GSE39501 dataset to screen for co-expression modules. In the course of the WGCNA, we first clustered the POI and Normal groups in GSE39501 using cluster tree and annotated the grouping information (without setting cut height). Subsequently, we set a screening criterion of 0.9 to determine the optimal number of modules. DEGs were clustered in 14 modules: MEcyan, MEdarkgrey, MEdarkred, MEorange, MEred, MEdarkorange, MEmidnightblue, MEpurple, MEdarkturquoise, MEgrey60, MElightyellow, MElightcyan, MEblue, and MEmagenta (Fig. 7A). Subsequently, we performed another round of clustering of the DEGs, visualizing the relation between the genes and their corresponding new modules. Finally, according to the expression pattern of the module genes and the different grouping information of the GSE39501 dataset, we obtained 14 modules (MEcyan, MEdarkgrey, MEdarkred, MEorange, MEred, MEdarkorange, MEmidnightblue, MEpurple, MEdarkturquoise, MEgrey60, MElightyellow, MElightcyan, MEblue, and MEmagenta). The correlation between the two groups in GSE39501 is depicted in (Fig. 7B). Subsequently, we applied a module merge shear height of 0.2 to shear and combine modules with a merge shear height below 0.2 (Fig. 7C).

First, we conducted a subsequent analysis of DEGs in the 14 modules (MEblue, MEdarkgrey, MEdarkred, MEmidnightblue, MEred, and MEorange) (P < 0.05, absolute correlation value 0.5). Second, we crossed the ICRDEGs in the GSE39501 dataset and the DEGs included in the six modules and generated a Venn diagram (Fig. 7D–I) to obtain the modules. As shown in Fig. 7, we obtained seven ICRDEGs (*Cd200*, *Cd274*, *Cd28*, *Nrp1*, *Cd276*, *Cd40lg*, and *Cd47*) as hub genes for our subsequent study.

We annotated the human chromosome locations for the seven hub genes and visualized them in a circle (Fig. 7J). The chromosome locations are as follows: *CD28* on chromosome 2, *CD47* and *CD200* on chromosome 3, *CD274* on chromosome 6, *NRP1* on chromosome 10, *CD276* on chromosome 15, and *CD40LG* on chromosome X.

We performed a functional similarity analysis of the seven hub genes by calculating the GO terms using the R package GOSemSim package. The analysis included the identification of GO Term Set (sets of GO terms), measuring the Semantic similarity between the gene products and gene cluster. Subsequently, the results of the functional similarity analysis between the seven hub genes were visualized through a boxplot (Fig. 7K). The results showed that *Cd274* has the highest functional similarity with the other hub genes.

### Expression and correlation analyses of the hub genes in the dataset

**Fig. 8:**
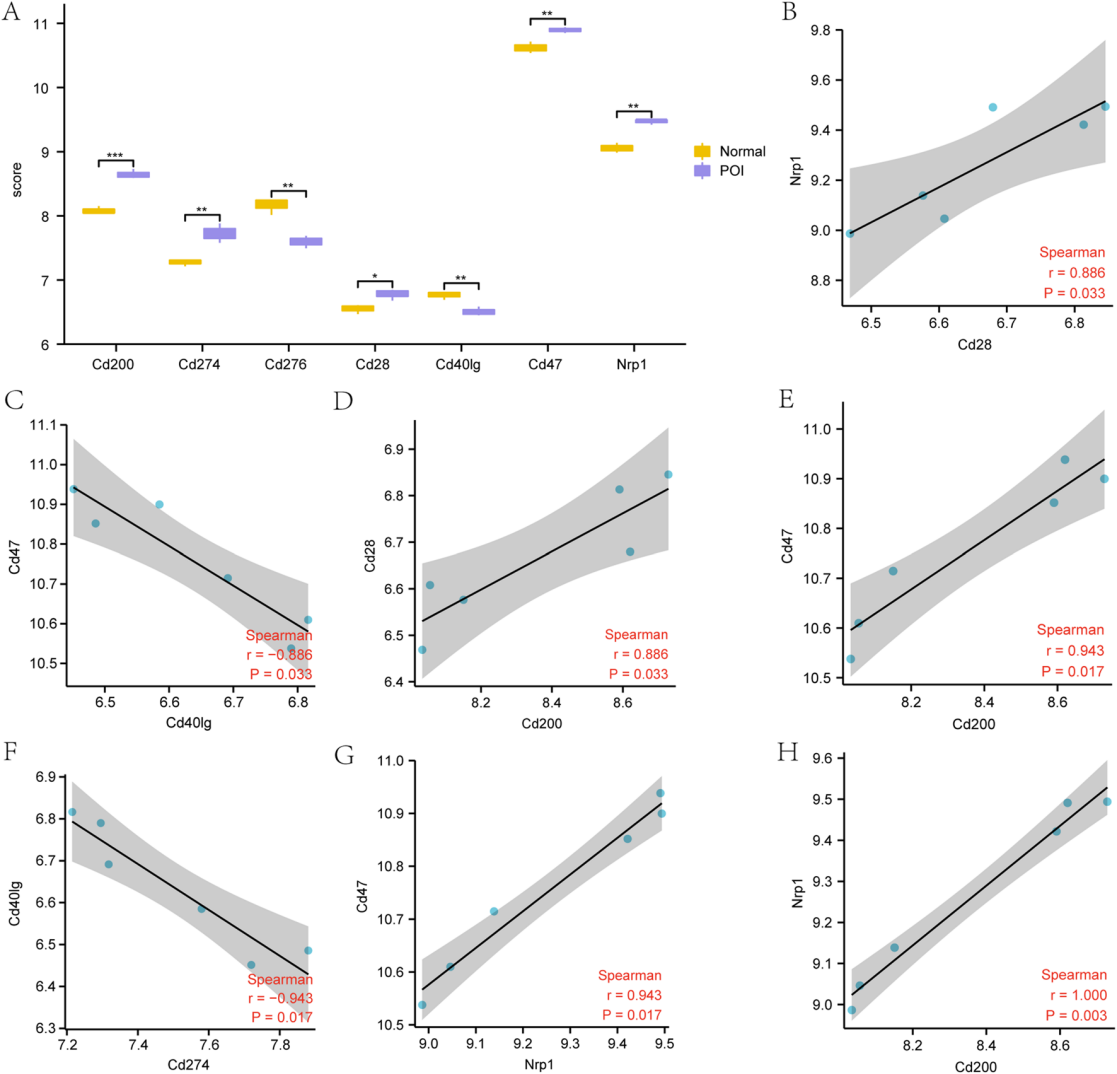
Differential expression and correlation analyses of the hub genes in the GSE39501 dataset. **A**. Comparative analysis of the expression levels of the hub genes in the GSE39501 dataset. B–H Scatter plots displaying correlation analysis results (CorResults) of the hub genes in the GSE39501 dataset. The symbol * indicates *P* < 0.05, which represents some statistical significance. The symbol * * indicates *P* < 0.01, representing high statistical significance. The symbol * * * indicates *P* < 0.001 and represents very high statistical significance. ICRDEGs: immune checkpoint-related differentially expressed genes. POI: premature ovarian insufficiency

To further explore the differences in expression of the hub genes in the POI dataset, we selected the seven hub genes screened by constructing the WGCNA network (*Cd200*, *Cd274*, *Cd28*, *Nrp1*, *Cd276*, *Cd40lg*, and *Cd47*) and further analyzed the correlation between the expression level in the GSE39501 dataset (Fig. 8A) and the Normal and POI groups. The results showed that the expression of hub gene *Cd200* in the GSE39501 dataset was statistically significant (P < 0.001). The expression levels of hub genes *Cd274*, *Cd276*, *Cd40lg*, *Cd47*, and *Nrp1* were significantly different between the Normal and POI groups (P < 0.01). Additionally, the expression levels of hub gene *Cd28* showed statistical significance in the Normal and POI groups (P < 0.05).

To further assess the correlation between the seven hub genes, we generated a heat map of the correlation between genes (Fig. 8B–H) and noted a statistically significant difference between *Cd47* and *Cd40lg* (*P* = 0.033). The correlation between *Nrp1* and *Cd200* showed a highly significant difference (*P* = 0.003).

To explore the differences in the expression of the seven hub genes in the POI dataset, we verified the differential expression of the genes among the Normal and POI groups, and the results are shown in Supplementary File Fig. S1.

### PPI and mRNA-miRNA mRNA-TF mRNA-RBP interaction networks

**Fig. 9:**
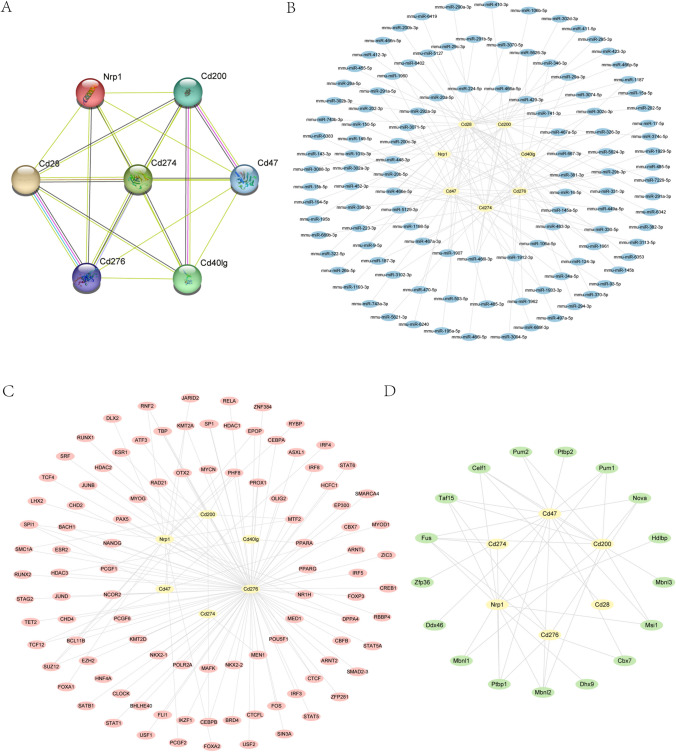
Protein–protein interaction (PPI) and mRNA-miRNA, mRNA-TF, mRNA-RBP interaction networks. **A**. The PPI network of the ICRDEGs. **B**. The hub genes-miRNA interaction network. **C**. The hub genes-transcription factor interaction network. **D**. The hub genes-RBP interaction network. Yellow ellipse depicts mRNA. The blue ellipse depicts miRNA. The pink ellipse depict transcription factors. The green ellipse depicts the RBP. POI: premature ovarian insufficiency

We used the STRING database for seven ICRDEGs (*Cd200*, *Cd274*, *Cd28*, *Nrp1*, *Cd276*, *Cd40lg*, and *Cd47*) for the PPI analysis, taking the minimum intercorrelation coefficient greater than 0.400 (minimum required interaction score: low confidence [0.150]) as the standard. The resulting PPI network was visualized and mapped using Cytoscape software (Fig. 9A).

We used the mRNA-miRNA data from the ENCORI and miRDB databases with seven ICRDEGs (*Cd200*, *Cd274*, *Cd28*, *Nrp1*, *Cd276*, *Cd40lg*, and *Cd47*) for prediction of the interacting miRNAs. Subsequently, the intersection of the two database results was visualized by drawing the mRNA-miRNA interaction network using Cytoscape software (Fig. 9B). Our mRNA-miRNA interaction network is composed of seven hub genes (mRNA) (*Cd200*, *Cd274*, *Cd28*, *Nrp1*, *Cd276*, *Cd40lg*, and *Cd47*), and a total of 112 miRNA molecules, resulting in 121 mRNA-miRNA interaction pairs (Table S2).

We searched for the TFs bound to the hub genes using the CHIPBase database (version 3.0). The mRNA-TF interaction network was then visualized using Cytoscape software (Fig. 9C), resulting in interaction data for six hub genes (mRNA) (*Cd47*, *Cd200*, *Cd274*, *Cd276*, *Nrp1*, and *Cd40lg*) and 101 TFs, forming 124 mRNA-TF interaction pairs (Table S3).

We used the mRNA-RBP data from the ENCORI database to predict interactions with seven ICRDEGs (*Cd200*, *Cd274*, *Cd28*, *Nrp1*, *Cd276*, *Cd40lg*, and *Cd47*). The database results were then visualized as an mRNA-RBP interaction network using Cytoscape software (Fig. 9D). Our mRNA-RBP interaction network is composed of six hub genes (mRNA) (*Cd200*, *Cd274*, *Cd276*, *Cd28*, *Cd47*, and *Nrp1*) and 17 RBP molecules, forming a total of 42 mRNA-RBP interaction pairs (Table S4).

### Immune infiltration analysis of the POI dataset

**Fig. 10:**
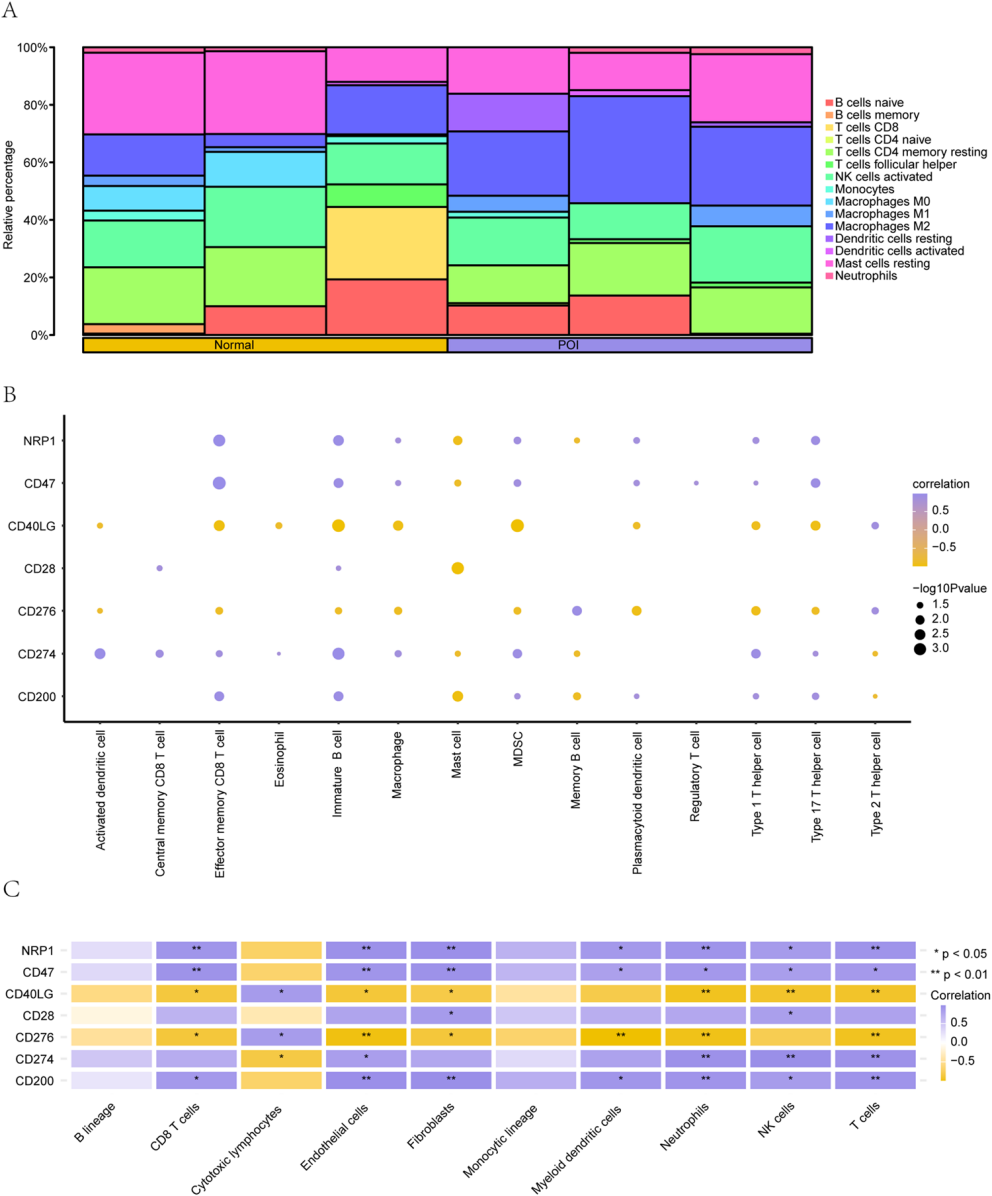
Analysis of immune infiltration in the GSE39501 dataset (CIBERSORT). **A**. Bar graph of immune infiltration results of 22 immune cells. **B–C**. Heat map of the correlation analysis of ICRDEGs and immune cell expression. The symbol * indicates *P* < 0.05, which represents statistical significance. The symbol ** indicates *P* < 0.01, representing high statistical significance. ICRDEGs: immune checkpoint-related differentially expressed genes. POI: premature ovarian insufficiency. In the heatmap, circles indicate genes with a positive correlation with the infiltration abundance of immune cells. Larger circles indicate stronger correlations. Yellow circles represent a negative correlation between genes and infiltration abundance of immune cells, with larger circles indicating a stronger correlation

We used the CIBERSORT algorithm to calculate the correlation between the expression profile data of the Normal and POI groups from 22 immune cells in the GSE39501 dataset. Based on the results of the immune infiltration analysis, we plotted the infiltration of 22 immune cells in each sample of the GSE39501 dataset (Fig. 10A) using bar charts.

We analyzed the differences in gene expression in 22 immune cells among the Normal and POI groups and generated a group comparison diagram. The results are shown in Supplementary File Fig. S1.

We used the ssGSEA algorithm to calculate the correlation between the expression profile data of the Normal and POI groups. Based on the results of the immune infiltration analysis, we identified a correlation between seven hub genes (*Cd200*, *Cd274*, *Cd28*, *Nrp1*, *Cd276*, *Cd40lg*, and *Cd47*) (Fig. 10B) (P < 0.05). In the GSE39501 dataset, a strong correlation was observed between the abundance of infiltrating immune cells, immature B cells, and *CD40LG*.

We assessed the correlation between the expression profiles of the Normal and POI groups in the GSE39501 dataset (Fig. 10C) using the MCPCounter algorithm. MCPCounter showed that the *NRP1*, *CD47*, *CD40LG*, *CD28*, *CD276*, *CD274*, and *CD200* genes were significantly positively associated with a variety of immune cells.

## Discussion

In a previous study, Wagner et al. used single-cell sequencing to demonstrate macrophages and T cells account for 0.5% of the ovarian cortex [24]. These immune cells can be involved in abnormal ovarian functions, such as abnormal ovulation [25], reduced oocyte number [26], destruction of the ovarian periodic angiogenesis system [27], and increased follicular atresia [25]. However, at present, the mechanism of ovarian dysfunction caused by immune-related reasons is not clear, and there is no specific immune diagnostic index for early diagnosis of ovarian failure. Therefore, it is necessary to elucidate the immune mechanisms related to ovarian disease. In this study, we first identified DEGs and immune checkpoint genes associated with POI. We then analyzed their related pathways and biological functions, and used WGCNA to select seven core genes. We finally performed a series of analyses of core genes to analyze and predict their associated potentially pathogenic mechanism.

There are 14 ICRDEGs, according to GO function enrichment analysis, ICRDEGs were mainly involved in the regulation immunity and inflammation biological process. The four pathways enriched in KEGG analysis were all involved in the T cell receptor signaling pathway. T cell-mediated immune responses can be divided into T cell activation, proliferation, and differentiation. T cell activation requires co-stimulation of dual signals, the first signal comes from the antigen and requires the interaction and binding of the major histocompatibility complex-antigen peptide complex on the antigen-presenting cell surface with T cell receptor, which ensures antigen specificity of the immune response. The second signal is a costimulatory molecule, which ensures that the immune response occurs only under the desired conditions. The dual-signal stimulation mode of T cell activation is a fail-safe mechanism, and the second signal ensures the correct timing and site of initiating T cell response. The lack of a second signal can keep autoreactive T cells in a nonresponsive state, which favors autoimmune tolerance and prevents the activation of autoreactive T lymphocytes. *CD28* and *CTLA-4* are involved in the regulation of T cell activation. Studies have shown that genetic defects in *CTLA-4* lead to *CD28*-mediated severe autoimmunity [28]. Based on our analysis, we can suspect POI-related immune checkpoint genes are involved in abnormal T cell activation, potentially contributing to the occurrence of disease. This insight provides a promising direction for future investigations into the relationship between T cell activation and POI.

According to the GSVA results, all genes in the GSE39501 datset were significantly enriched in the phosphatidylinositol 3-kinase-AKT pathway, MAPK pathway, TGFbeta pathway, WNT pathway, NOTCH pathway, HEDEGHOG pathway and TP53 pathway. Members of the phosphatidylinositol 3-kinase family has important functions in the immune system. PI3K is an important downstream effector of BCR signaling, further activating the expression of other genes involved in B cell proliferation, differentiation, and innate immune [29]. The MAPK pathway is a canonical inflammatory pathway and key regulator of the inflammatory response and innate immunity. The GSVA results showed that the IL-2 pathway isogene set showed differences between the POI and Normal groups. IL-2 is the result of T cell stimulation and further promotes T cell stimulation[30, 31]. The enrichment of the IL-2 pathway suggested an adaptive immune component to POI. These results indicate that the genes and pathways that were significantly different between the disease and normal group samples of the GSE39501 dataset involved both innate and adaptive immunity, as well as inflammatory responses.

The WGCNA-ICRDEG intersection identified seven core genes, *Cd200*, *Cd274*, *Cd28*, *Nrp1*, *Cd276*, *Cd40lg*, and *Cd47* as well as 112 miRNAs, 101 TFs, and 17 RBPs regulatory networks for the core genes. Expression and correlation analyses of core genes in the dataset showed that *CD200* was differentially expressed in the Normal and POI groups, and the correlation between *Nrp1* and *CD200* was significant in the POI dataset.

*CD200* (*OX-2*) belongs to the type I membrane glycoprotein and immunoglobulin superfamily [32] and is expressed in epithelial cells, endothelial cells, fibroblasts, lymphocytes, neurons, ovarian granulosa cells, downregulated myeloid cells (neutrophils, macrophages, and dendritic cells). Studies have shown that *CD200* regulates immune and inflammatory responses [33] by regulating *CD200R* to inhibit macrophage activation and transmit immunosuppressive signals to macrophages. Studies have also shown that macrophages are ubiquitous in the female reproductive system, playing physiological roles in the female menstrual cycle and participating in the regulation of the pituitary–gonadal axis [34]. The proportion of macrophage subsets in old mice is significantly increased [26] when compared with that in younger mice. Thus, we need conduct further experimental studies to verify the mechanism by which *CD200*-mediated POI increases the susceptibility to ovarian immune diseases due to disruptions in macrophages physiological function.

The correlation analyses revealed a significant correlation between *Nrp1* and *CD200*. *Nrp1* is a transmembrane glycoprotein with important roles in embryonic tissue development, angiogenesis, and tumor metastasis [35]. Both *Nrp1* and *CD200* function as negative regulators of the immune response. While their roles in reproductive functions are less explored, the potential involvement of both in the immune pathogenesis of POI requires further research.

The negative regulation of the immune response and physiological function by the core genes previously mentioned serves to prevent immune damage to body tissue and reduce immune activation. Any abnormality in these regulatory mechanisms can lead to unpredictable immune damage. Although the exact causes of POI are still unclear, numerous experiments have demonstrated the involvement of immune system disorders in its pathogenesis [36]. The immune checkpoint molecules discussed previously could serve as focal points for future investigations into immune-related genes.

The results of the immune infiltration analysis showed a strong correlation between the infiltration abundance of immature B cells and *CD40LG* in the ssGSEA algorithm, indicating that *CD40LG* may play an important role in regulating or affecting the infiltration abundance of immature B cells. MCPCounter showed that the *NRP1*, *CD47*, *CD40LG*, *CD28*, *CD276*, *CD274*, and *CD200* genes were positively associated with a variety of immune cells, implying that the core gene plays an important role in the immune regulation of POI, although the specific regulatory mechanism requires further study.

## Conclusions

The results of this study indicate that T cell-mediated immune and inflammatory responses may be involved in the pathogenesis of premature ovarian aging. Furthermore, there is a need for future investigation into the effects of the T cell receptor signaling pathway and immune checkpoint molecules *CD200* and *NRP1* on the ovarian immune system.

### Supplementary Information

Below is the link to the electronic supplementary material.

Supplementary file1 (PDF 7 KB)

Supplementary file2 (PDF 8 KB)

Supplementary file3 (PDF 7 KB)

Supplementary file4 (PDF 6 KB)

Supplementary file5 (PDF 6 KB)

Supplementary file6 (PDF 6 KB)

Supplementary file7 (PDF 6 KB)

Supplementary file8 (PDF 6 KB)

Supplementary file9 (PDF 6 KB)

Supplementary file10 (PDF 6 KB)

Supplementary file11 (DOCX 13 KB)

Supplementary file12 mRNA-microRNA interaction network nodes. (XLSX 11 KB)

Supplementary file13 mRNA-transcription factor interaction network nodes. (XLSX 11 KB)

Supplementary file14 mRNA-RNA binding protein interaction network nodes. (XLSX 9 KB)

## Data Availability

Data are available in a public, open access repository. Data are available on reasonable request. All data relevant to the study are included in the article or uploaded as supplemental information.The datasets (GEO data) for this study can be found in the GEO (https://www.ncbi.nlm.nih.gov/geo/query/acc.cgi?acc=GSE39501).
