# Supplementary figures and images for: Bioinformatic analysis of the role of immune checkpoint genes and immune infiltration in the pathogenesis and development of premature ovarian insufficiency
Source: J Assist Reprod Genet. 2024 May 2;41(6):1619–35. doi: 10.1007/s10815-024-03120-x (PMC11224201; doi:10.1007/s10815-024-03120-x)

GSE39501

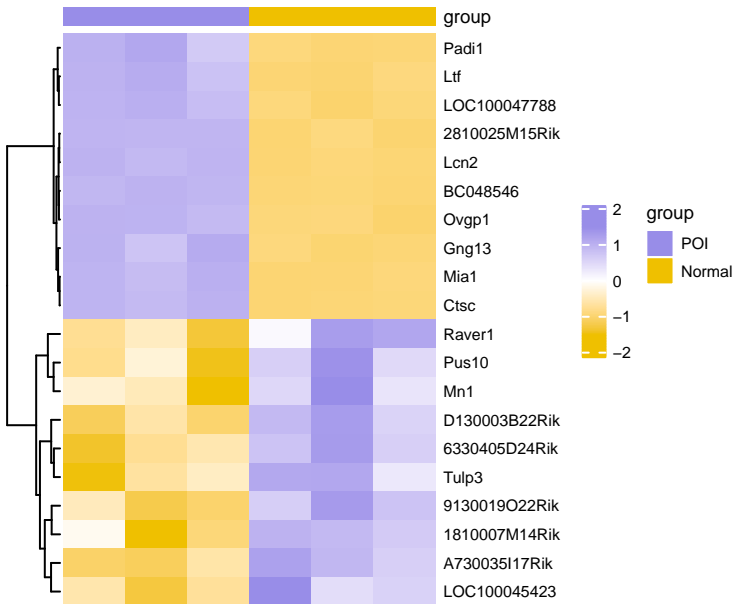

Supplement: Supplementary file 2 — Supplementary file2 (PDF 8 KB) [file 10815_2024_3120_MOESM2_ESM.pdf]

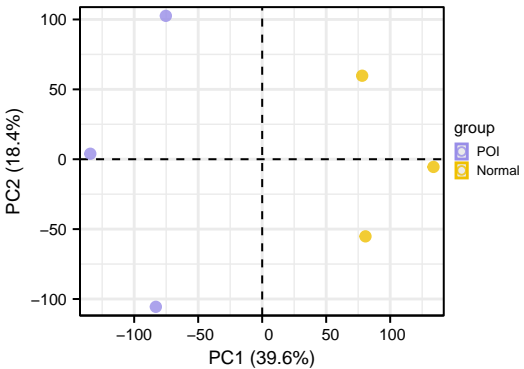

Supplement: Supplementary file 3 — Supplementary file3 (PDF 7 KB) [file 10815_2024_3120_MOESM3_ESM.pdf]

# GSE39501

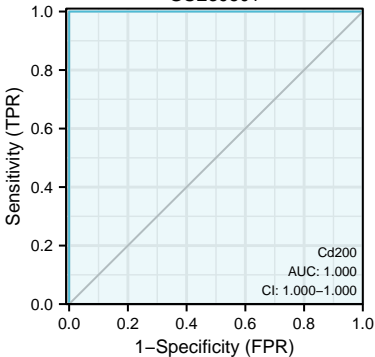

Supplement: Supplementary file 4 — Supplementary file4 (PDF 6 KB) [file 10815_2024_3120_MOESM4_ESM.pdf]

# GSE39501

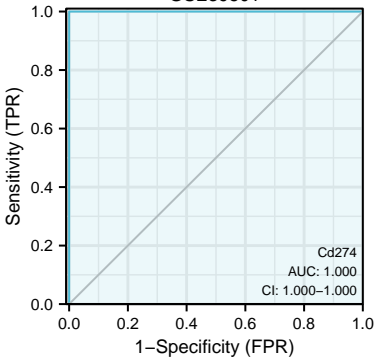

Supplement: Supplementary file 5 — Supplementary file5 (PDF 6 KB) [file 10815_2024_3120_MOESM5_ESM.pdf]

# GSE39501

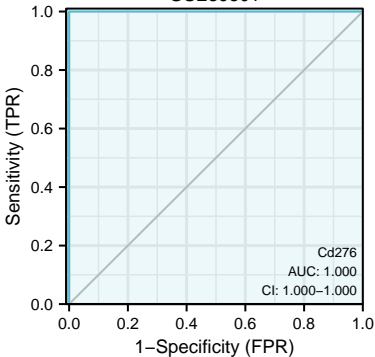

Supplement: Supplementary file 6 — Supplementary file6 (PDF 6 KB) [file 10815_2024_3120_MOESM6_ESM.pdf]

# GSE39501

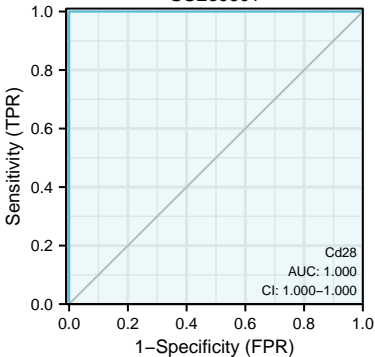

Supplement: Supplementary file 7 — Supplementary file7 (PDF 6 KB) [file 10815_2024_3120_MOESM7_ESM.pdf]

# GSE39501

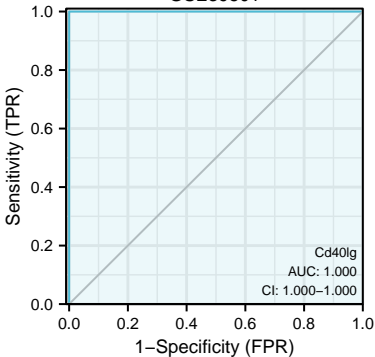

Supplement: Supplementary file 8 — Supplementary file8 (PDF 6 KB) [file 10815_2024_3120_MOESM8_ESM.pdf]

# GSE39501

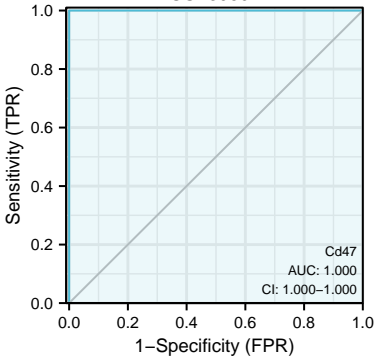

Supplement: Supplementary file 9 — Supplementary file9 (PDF 6 KB) [file 10815_2024_3120_MOESM9_ESM.pdf]

# GSE39501

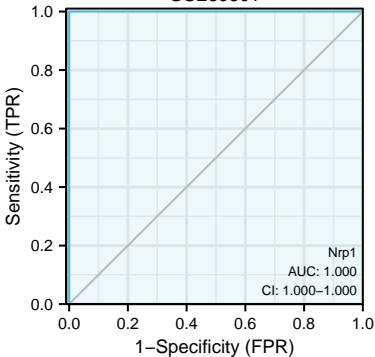

Supplement: Supplementary file 10 — Supplementary file10 (PDF 6 KB) [file 10815_2024_3120_MOESM10_ESM.pdf]
