# Supplementary material for: Bioinformatic analysis of the role of immune checkpoint genes and immune infiltration in the pathogenesis and development of premature ovarian insufficiency
Source: J Assist Reprod Genet. 2024 May 2;41(6):1619–35. doi: 10.1007/s10815-024-03120-x (PMC11224201; doi:10.1007/s10815-024-03120-x)
Supplement: Supplementary file 11 — Supplementary file11 (DOCX 13 KB) [file 10815_2024_3120_MOESM11_ESM.docx]

### table S1. Immune Checkpoint-related genes.

| Immune Checkpoint-related differentially expressed genes | | | | | |
| --- | --- | --- | --- | --- | --- |
| BIRC3 | B7-H4 | CD200R1 | CTLA4 | PD1LG2 | TNFSF18 |
| G0S2 | LILRB2 | CD244 | HAVCR2 | PDCD1 | TNFSF4 |
| CCR7 | TIM3 | CD27 | HHLA2 | TIGIT | TNFSF9 |
| CPS1 | CD47 | CD274 | ICOS | TMIGD2 | VTCN1 |
| CLEC7A | CD137 | CD276 | ICOSLG | TNFRSF14 | CXCL1 |
| LILRB1 | CD70 | CD28 | IDO1 | TNFRSF18 | CXCL10 |
| CCR2 | ADORA2A | CD40 | IDO2 | TNFRSF25 | CXCL11 |
| HLA-DOB | BTLA | CD40LG | KIR3DL1 | TNFRSF4 | CCL8 |
| CD1E | BTNL2 | CD44 | LAG3 | TNFRSF8 | CCL13 |
| PD-L1 | C10orf54 | CD48 | LAIR1 | TNFRSF9 | CCL18 |
| PD-1 | CD160 | CD80 | LGALS9 | TNFSF14 | CD38 |
| B7-H3 | CD200 | CD86 | NRP1 | TNFSF15 | KLRD1 |
| FCRL4 |  |  |  |  |  |
