# Supplementary material for: Bioinformatic analysis of the role of immune checkpoint genes and immune infiltration in the pathogenesis and development of premature ovarian insufficiency
Source: J Assist Reprod Genet. 2024 May 2;41(6):1619–35. doi: 10.1007/s10815-024-03120-x (PMC11224201; doi:10.1007/s10815-024-03120-x)
Supplement: Supplementary file 1 — Supplementary file1 (PDF 7 KB) [file 10815_2024_3120_MOESM1_ESM.pdf]

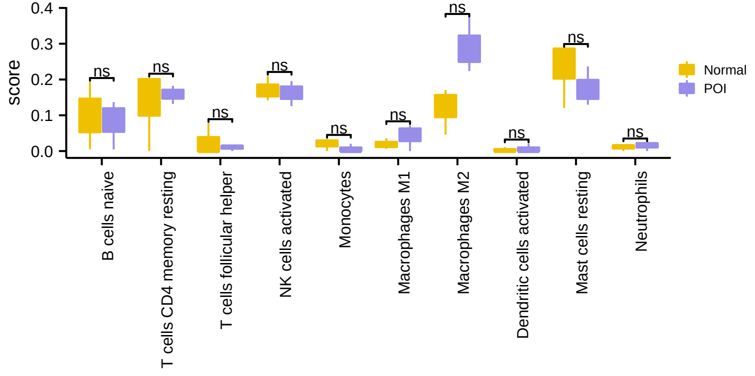

score
0.4
0.3
0.2
0.1
0.0
ns
B cells naive
T cells CD4 memory resting
T cells follicular helper
NK cells activated
Monocytes
Macrophages M1
Macrophages M2
Dendritic cells activated
Mast cells resting
Neutrophils
Normal
POI
